# Support Possibilities for 3D Scanning of Forging Tools with Deep and Slim Impressions for an Evaluation of Wear by Means of Replication Methods

**DOI:** 10.3390/ma13081881

**Published:** 2020-04-17

**Authors:** Marek Hawryluk, Zbigniew Gronostajski, Jacek Ziemba, Marta Janik, Piotr Górski, Miłosz Lisowski

**Affiliations:** 1 Welding and Metrology, Department of Metal Forming, Wroclaw University of Science and Technology, 50-370 Wroclaw, Poland; zbigniew.gronostajski@pwr.edu.pl (Z.G.); jacek.ziemba@pwr.edu.pl (J.Z.); piotr.gorski@pwr.edu.pl (P.G.); milosz.lisowski@gmail.com (M.L.); 2 Mahle Poland, 63-700 Krotoszyn, Poland; marta.janik@pl.mahle.com

**Keywords:** 3D scanning, replication method, imprint from flexible mass, extrusion die for valves, wear, loss of material

## Abstract

This article discusses the problems related to the use of non-contact 3D scanning techniques and their support by means of replication methods for the analysis of the geometrical changes in deep tool impressions used for the forward extrusion of valve-type elements assigned for motor truck engines. The 3D scanning method, despite its unquestionable advantages, also has certain limitations, such as scanning the inner surfaces of deep cavities. This is caused by the fact that the larger the angle between the reflected laser light and the normal direction to the measured surface, the larger the area covered for the analysis, yet at the same time, the higher the measurement error. The authors performed an analysis of the geometrical loss of the tools as well as the corresponding replication masses, together with a discussion of the results related to minimization of the measuring errors. For the analyzed tool, the maximum angle during direct scanning was 40 degrees, which unfortunately does not enable an analysis of the entire pattern, while for larger angles, it is necessary to make the measurement by indirect scanning, i.e., by replicating the cavity imprint of the tool. Therefore, for a given geometry, the reflection angle should be determined individually.

## 1. Introduction

**Figure 1:**
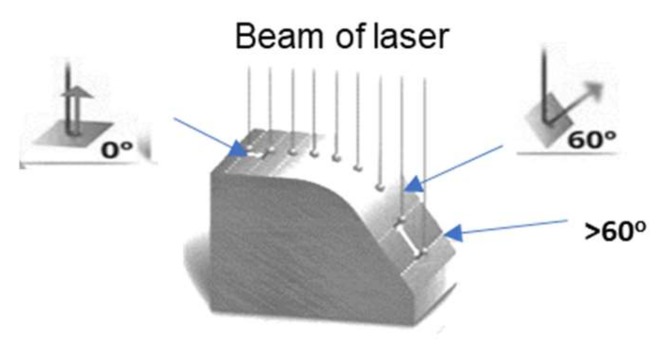
Example of the operation of a scanning angle filter for various angle values.

The coordinate measurement technique is at present commonly applied in e.g., the forging industry [1,2], for the measurement of both forged elements–forgings [3,4] (with different degrees of geometry complexity) as well as the tools shaping those products. For the measurements, not only the traditional measurement methods based on the classic measurement equipment, but also modern non-contact measurement methods based on 3D scanning are applied [5,6,7]. Such an approach has been demonstrated in the studies of Hawryluk and Ziemba [8,9] for the wear analysis of die inserts. It should, however, be pointed out that not all forging tools have a geometry which can be analyzed with the use of 3D scanning methods. Often, in precision die forging processes, especially in operations of forward extrusion of long slim forgings, forging dies with deep impressions and complicated geometries are used, which makes it impossible to apply 3D laser scanning [10]. Also, at times, during the 3D scanning of especially complicated inner surfaces, e.g., deep and slim cavities constituting the working impressions of the tools, but also other elements/objects, excessive measurement errors may occur, which results in the necessity to consciously limit the measurement field by the operator of the scanner [11,12,13,14]. In turn, in the case when, despite a relatively complicated inner geometry, it is possible to collected a cloud of points covering the whole such surface during the physical scanning, it might turn out that the results for some of the scanned areas are still burdened with a hard to estimate measurement error [15,16]. This results from the fact that the modern software for measurement data collection for linear 3D scanners, in order to increase the scanning accuracy, has different concepts of eliminating the disadvantageous measurement phenomena, so that more precise results can be obtained through filtering of the data burdened with excessive errors [17]. One of the concepts of measurement point filtration applying the real time quality meshing algorithm [18] makes it possible to control the process in real time, providing the scanner’s operator with the possibility of influencing the error by orienting the laser beam in respect of the scanned surface (Figure 1). This technique is based on analyzing the angle between the laser beam and the normal direction to the scanned surface. On the basis of the calculated angle value during the scanning process, the software automatically rejects the data with the value above a certain predetermined threshold [19,20]. This way, only the best quality data are collected.

Conventionally, the scanner’s maximal angle value in the software is set to 55°. Such a value has been determined based on the error acceptable in the given/universal application, with a simultaneous minimization of the measurement area elimination. In turn, in order to increase the measurement accuracy, this angle should be reduced, which, unfortunately, at the same time, causes a reduction of the measurement area. In this case, the laser-scanned data contains inaccurate data points at spatial discontinuities (object edges). These inaccurate points, known as mixed pixels, are usually removed from the data before geometric modeling or other subsequent processes. Removing points on the edges of the objects introduces an error in their geometry, and their dimensions extracted from the data, such as the width and the height, may be smaller or larger than the actual values, depending on the given geometry. In such cases, losses due to the removal of points on the edges may exceed the tolerances for measurement accuracy specified in the inspection instructions [21,22,23].

At present, owing to the continuous progress in science and new technologies, various methods are being developed whose aim is to eliminate the necessity of cutting the examined objects assigned for the measurement and, instead, perform non-destructive tests (NDT) applying, e.g., methods based on the replication technique [24,25]. These techniques are based on the use of various synthetic substances [26], mainly different types of resins, especially epoxy resins, polyurethane rubbers [27], silicones, but also other materials (gypsum, liquid glass, wax, plasticine, soft metals, etc.) characterizing good formability, castability, and filling, and after their hardening or solidification [28], in being easily removed from the measured elements. They also provide a good and durable representation of the filled mould [29,30,31]. Interest in specialized material properties has been growing in technologies of shape representation, mainly prosthetics, e.g., in implantology, stomatology, etc. [32,33]. A further development of replication techniques has led to intensified studies of the use of materials which are both well-formable and able to fill complicated shapes, and at the same time, characterized by a minimized shrinkage and plastic deformation after solidification, and which could be easily removed from elements of a complicated geometry with a contraction [34,35]. Replication mass impressions are characterized by high mapping accuracy of up to 1 micrometer [36]. Additionally, big elastic deformations with relatively high hardness of such materials have enabled precise shape representation and removal of the representing material from a geometry of negative inclinations [37].

All applications share the use of different measurement methods of geometry representation, including non-contact 3D scanning techniques, which are, as it were, predestined for the measurements of replication masses, for elements constituting e.g., forging and extrusion tools characterized by deep and slim working impressions with a relatively complicated geometry [10].

The goal of the study is to determine the effect of the measurement errors (dependent on the angle between the laser scanner beam and the normal direction to the scanned surface) during the wear analysis of forging dies with deep and slim impressions as well as to support the measurements of this type by way of using replication techniques (high accuracy of mapping), which, in combination with the 3D reverse scanning method, make it possible to precisely represent and determine the actual state of worn forging tools.

## 2. Materials and Methods

**Figure 2:**
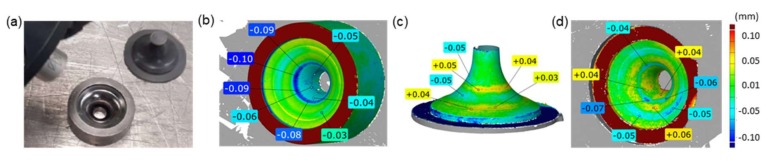
The idea of the developed method of die wear analysis: (**a**) 3D scanning—measurements of the die and the replication mass imprint, (**b**) result of a direct measurement of the die burdened with errors caused by the imperfection of the scanning techniques, (**c**) results of a measurement through scanning of the replication mass imprint in the die, (**d**) result of a measurement (scanning) showing the real surface state of a worn tool without errors obtained by way of superimposing a reversed replica image onto a CAD model.

The approach proposed by the authors (Figure 2) consists in developing a new, useful, and reliable measurement method (in the field of non-destructive testing NDT).

**Figure 3:**
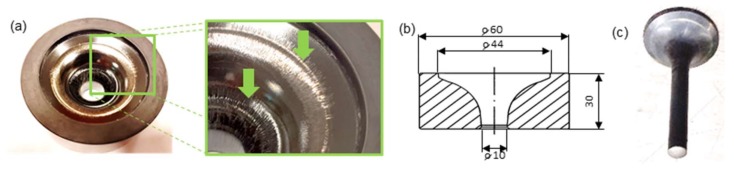
A selected, exemplary forging tool for the analyzed die forging process (**a**) removed from the production process, with visible wear for a die insert used in roughing, (**b**) key geometrical dimensions, (**c**) photograph of an exhaustion valve forging assigned for motor truck engines.

This method consists in combining 3D scanning techniques with the replication mass imprint technique for an analysis of the geometrical changes in complicated inner surfaces of tools (used for the extrusion of engine valve forgings), which are difficult to determine by means of other methods, e.g., measurements with the use of a CMM (coordinate measuring machine), etc. The tests were performed on worn-out forging tools (Figure 3a), characterizing deep and relatively slim working impressions (which significantly complicated their measurement by means of typical techniques, as shown in Figure 3b), and used to produce axisymmetric forgings constituting exhaustion valves for motor trucks, in the process of two-operation precise hot forging. In order to represent and analyze the state of their surface through a comparison, independent 3D scanning procedures of both the tool impressions and the replication mass imprints were proposed. Therefore, the research was divided into two stages:

- Scanning of the dies’ working impressions and an analysis of the effect of the laser beam angle with respect to the scanned surface on the error size;
- Scanning of the replication mass imprints of the die working impressions and measurement support to obtain proper results showing the actual state of the tools.

A forging of a valve (Figure 3c) is made of difficult-to-shape chromium-nickel steel and is a key responsible element of the engine, and additionally, due to restricted requirements for the automotive industry, it requires special supervision of its production process. That is why, in the industrial process of precise hot forging, stable and repeatable conditions have to be ensured. In such a case, very important are the requirements related to the dimensional and shape accuracy as well as the parameters of the surface layer (quality of the coating application, hardness, etc.) for a set of forging tools consisting of a die and a stamp. The tools used in the selected process, as a result of their wear (extreme working conditions: cyclic mechanical loads reaching 1500 MPa and thermal loads of 1000 °C), undergo destruction mainly through intensive abrasive wear as well as plastic deformations caused by high temperatures, which result in local tempering of the working impression surfaces. The wear of forging tools (Figure 3a) causes a change in the geometry of the produced object. Also, all the tool surface defects (cracks, losses, scratches, etc.) are represented on the forged product, thus affecting the quality of the ready object. So, in order to increase their durability, forging tools undergo additional thermo-chemical treatments, e.g., through the use of surface engineering techniques, such as hybrid layers. Usually, an analysis of the surface state of such tools, due to their unique geometry, requires their being cut, which of course means a permanent removal of such a tool from the further production.

In the analyzed case, the tools were made of W360 steel and then underwent standard thermal treatment, together with the application of two types of hybrid layers: nitriding + ALWIN and nitriding + BIGAAN, in order to increase their durability. Tools without hybrid layers characterize in relatively low durability, at the level of about 500 forgings. In turn, after proper coatings have been applied, the time of their operation is prolonged a few times (even up to 2500 items). After the finishing mechanical treatment, the quality of the impression’s surface, according to the production technology, achieves the roughness parameter at the level from about 650 HV (core material) to as much as 2000 HV (surface layer).

The tests were divided into two stages. The first included a direct measurement with the use of the 3D scanning technique applied on a new as well as worn die working impression, in order to evaluate the effect of the angle between the laser beam and the normal direction to the scanned surface on the quality of the obtained products.

The second stage was constituted by 3D scanning of the imprints made from a replication mass for the analyzed dies, in order to determine the precision of the impression’s representation. The performed tests at this stage made it possible to verify the 3D reverse scanning method as well as determine the actual state of the analyzed worn-out forging tools, together with an analysis of the volumetric loss of the material.

**Figure 4:**
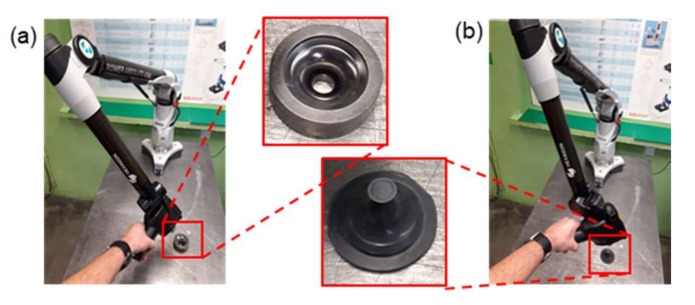
Test stand with a measuring arm ROMER AbsoluteArm 7520si integrated with a linear scanner RS3 for the measurements of: (**a**) tools, (**b**) replicas.

A measuring arm ROMER Absolute ARM 7520si (Hexagon Manufacturing Intelligence, Cobham, Great Britain) integrated with an RS3 scanner, together with the Polyworks software (2015 IR2, build 3435, InnovMetric, Quebec City, QC, Canada) and the measuring data filtering technology Real Time Quality Meshing (InnovMetric, Quebec City, QC, Canada) were applied in the tests. For the purposes of the investigations, a laboratorial test stand was constructed (Figure 4), in which a linear laser scanner integrated with the arm was used, which made it possible to collect up to 460,000 points/s for 4600 points on the line with the linear frequency of 100 Hz. The accuracy of the integrated scanning system SI according to the standard B89.4.22 equals 0.053 mm.

## 3. Results and Discussion

### 3.1. Scanning of the Working Impressions of Dies and Analysis of the Effect of the Laser Beam Angle with Respect to the Scanned Surface on the Error Size

**Figure 5:**
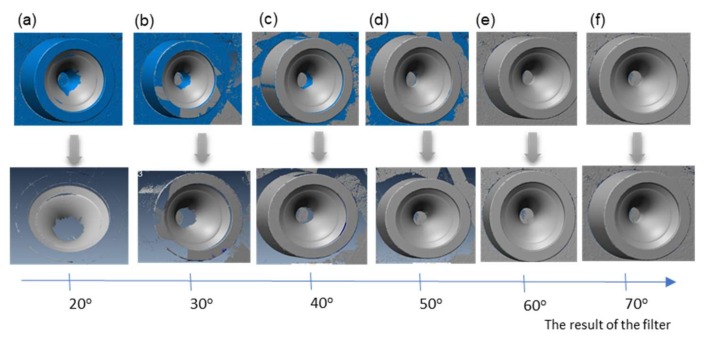
Effect of the change of the value of the angel between the normal direction and the reflected laser light in the process of filtering the measurement data on the change in the measurement area (blue-omitted area, grey-analyzed area: (**a**) up to 20°, (**b**) up to 30°, (**c**) up to 40°, (**d**) up to 50°, (**e**) up to 60°, (**f**) up to 70° for a new tool.

The first stage of the research consisted in evaluating the possibility of direct 3D scanning of the analyzed die impressions. Below (see Figure 5), we can see a simulation of the effect of a change in the filtering of the same measurement data through a determination of the maximal value of the angle between the normal direction and the reflected laser light during the 3D scanning of the new tool on the obtained measurement area (in the analyzed case, visible in the form of a reduced possibility to measure a deep cavity).

On the basis of the analysis (Figure 5), we can state that, for the programmed filtering of the measurement data by way of changing the value of the angle between the normal direction and the reflected laser light, when this value is minimized (e.g., to 20°), the measurement area possible for analysis becomes drastically reduced (Figure 5a). Thus, it becomes impossible to analyze the central part of the tool (inner surface of the deep cavity, entirely omitted, which visually resembles an unscanned surface). In turn, with a big difference up to 70°, it is possible to obtain the measurement data from the whole inner surface of the detail. It should, however, be emphasized that, this way, the additional area which was chosen for the analysis in the case shown in Figure 5f will be burdened with a big error.

**Figure 6:**
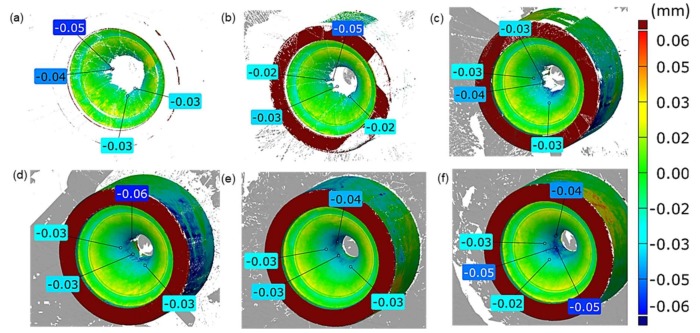
Measuring errors resulting from the change in the angle value between the normal direction and the reflected laser light in the process of filtering the measurement data affecting the change of the area obtained for the analysis: (**a**) up to 20°, (**b**) up to 30°, (**c**) up to 40°, (**d**) up to 50°, (**e**) up to 60°, (**f**) up to 70°^.^

A confirmation of such a state are the results presented in Figure 6, which shows the effect of a change in the angle’s acceptable value (between the normal direction and the reflected laser light in the process of filtering the measurement data) on the change of the area taken for the analysis, as well as the measuring errors in respect of an unworn tool (CAD model).

Analyzing the measurement data compilation presented above (Figure 6), we can notice that an increase of the acceptable angle value results in an increase of the measurement area taken for the analysis (deep and narrow, as well as slim cavity), in which, at the same time, the error becomes larger, and it is especially visible in the cavity. In turn, the visible yellow external ring (small error at the level of +0.02 mm) is probably the effect of the difference between the CAD model and the new tool made of metal. On this basis, we can state that, for the analyzed die geometry, in the inner section of the cavity, errors are visible, and their value can reach as much as 0.08 mm (for the angle of 70°, Figure 6f). In the analyzed case, the acceptable tolerance for the precision in producing the die’s geometrical features equals 0.03 mm. On this basis, in the analysis of the effect of the change in the angle value between the normal direction and then reflected laser light in the process of filtering the measurement data on the change in the area obtained for the analysis and the measuring errors, the angle 40° was assumed as the critical value (Figure 6c), in which case, the developing errors are acceptable in the analyzed application. However, assuming such a value makes it impossible to analyze the whole inner area of the die impression. That is why the use of the 3D scanning technique for such type of elements for the assumed tolerance, due to the occurrence of errors, may turn out difficult or even pointless. The remaining tools with hybrid layers which underwent the analysis were scanned with the standard angle at the level of 55°, in order to estimate the measurement errors (left column in Figure 8a). And so, it can be assumed that, for the analyzed tools, as a result of their wear (increasing material loss), the obtained results constitute a combination of the actual wear and an excessive error resulting from the imperfection of the applied methods of scanning deep and slim cavities.

### 3.2. Scanning of Replication Mass Imprints of Die Working Impressions—Measurement Support to Obtain Proper Results Showing the Actual State of the Tools

**Figure 7:**
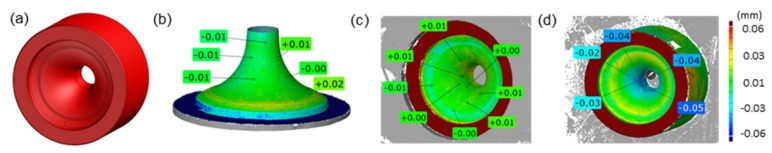
View of: (**a**) CAD model of nominal geometry, (**b**) coloured map of deviations of the die replica from the CAD model, (**c**) coloured map of deviations of the reverse scan of the replica from the CAD model (small errors pointing to high accuracy), (**d**) coloured map of deviations of the die’s 3D scan from the CAD model (for the angle value of up to 55°—default settings).

Obtaining reliable measurement data which fully illustrates the real state of the working surfaces of the analyzed tools can be aided by the use of replication masses. The latter make it possible to make imprints of those die impressions which are “difficult” to measure by means of a non-contact measurement technique as well as to perform their 3D scanning. An advantage is the fact that masses of this type are elastic, repeatable, and reproducible, and they are also very easy to remove from the impression of a worn tool and thus make it possible to reflect the actual geometrical state of the impression. Additionally, the measurement of replicas prepared in this way, which are easily deformable, predestines them to be used in a non-contact method in the form of 3D scanning. An advantage of the application of the technique of replicating deep cavities is the obtaining of much more accurate measurement results, which has been demonstrated by the authors in the studies [10]. Also, in this case, as shown in Figure 7 in the compilation of scans of a new die and its replica, the obtained results encourage the use of replication masses as well as the 3D scanning technique, with the standard angle set at the level of 55°. The results presented in Figure 7 suggest that the use of scans of replication mass imprints (Figure 7b,c) in relation to the CAD model of a new tool (Figure 7a) is fully justified, as the obtained measurement errors are at the level of the measurement accuracy of the scanner, which at the same time constitutes a confirmation and verification of the proposed measuring method.

**Figure 8:**
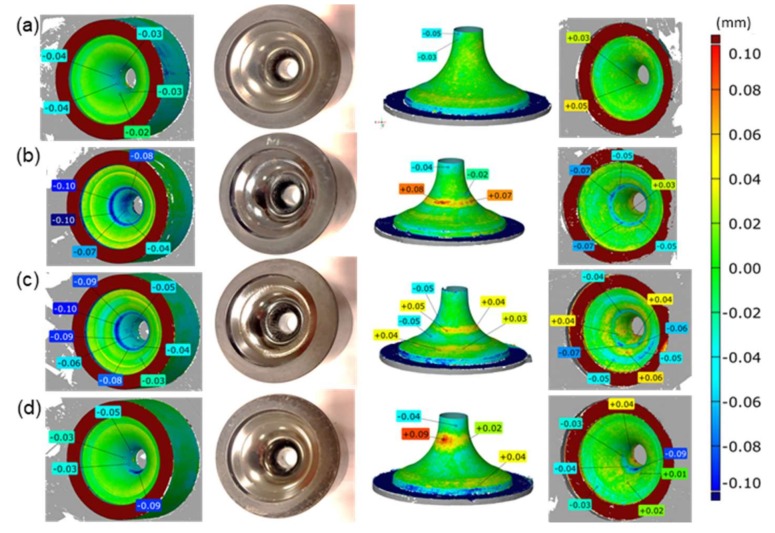
Compilation of the results of tool scanning, macro-photographs, 3D replica scans and a coloured mapof deviations for the dies obtained by way of reversing the 3D scan of the replica and comparing it to the nominal CAD scan (with standard setting of the angle, i.e., 55° for: (**a**) tools with an ALWIN coating after 1300 forgings, (**b**) with an ALWIN coating after 2400 forgings, (**c**) with a BIGAAN coating after 1090 forgings, (**d**) with a BIGAAN coating after 1900 forgings.

This case is different than that of the working impression of a new tool (Figure 7d), where the measurement errors (with the standard acceptable setting of the angle, i.e., 55°), especially in the central (deep) zone, are at the level of about 0.04–0.05 mm. So, with the assumption that, for a new-unworn tool, during the direct 3D scanning of its impression, the errors are at an unacceptable level, the wear of the tools is difficult to interpret, thus excluding the application of direct scanning of the impressions for a reliable evaluation of the surface state. Figure 8 shows a compilation of a coloured map of deviations for a 3D scan of a tool replica reflecting the actual state of the surface of worn forging tools for four selected dies covered with two types of coatings and with different degrees of wear. While analyzing the results presented in Figure 8 for the first (left) row and referring them to the macro-photographs of the tools (central row) as well as comparing them to the scans of the corresponding replication mass imprints, we can notice quite big differences, which suggests and at the same time confirms the fact that the 3D scanning of only the impressions with the analyzed geometry does not make it possible to obtain the desired results. However, with the use of the 3D replica scanning technique, there is a possibility to obtain a real image of the wear of forging tools used to produce valves.

In order to perform an in-depth analysis, it is necessary to use a technique which makes it possible to obtain a coloured map of deviations for the dies through reversing the 3D scans of the replicas and comparing them with the nominal CAD model, which is presented in the fourth column in Figure 8. When analyzing the compilation of the coloured map of deviations for the dies obtained by way of reversing the 3D scan of the replica and comparing it to the nominal CAD model, we can observe the actual state of the working surfaces of the selected tools, which points to their much lesser wear. In the comparison of the result from the first and fourth columns in Figure 8, we can see an error resulting from the direct scanning of only the tools, which caused “adding” of about 0.05–0.08 mm of material loss. What is interesting is that, comparing the results for the impression scans (Figure 8a) with the reversed replica scans (Figure 8d), we cannot see any significant differences. Only on the scans of the imprints can we notice both wear and material growth in the inner area of the impression (big cavity diameter: blue ellipsis).

**Figure 9:**
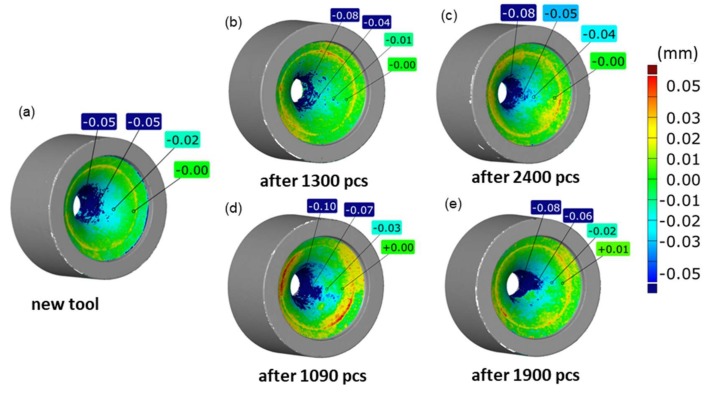
Comparison of the results of direct scanning of a die in relation to the scan of the replica from the dies (with standard setting of the angle, i.e., 55°) for: (**a**) new tool, (**b**) tools with an ALWIN coating after 1300 forgings, (**c**) with an ALWIN coating after 2400 forgings, (**d**) with a BIGAN coating after 1090 forgings, (**e**) with a BIGAAN coating after 1900 forgings.

In order to better reflect the differences resulting from the direct scanning of the tools (assumed to be error-burdened) and their replicas (correct measurements), an additional comparison was introduced, constituted by a map of deviations of the direct matrix scanning results (at the maximum allowable angle of 55°) in relation to the scan of the replica, which faithfully reproduces the geometry of the hole. In this way, you can assess the real errors of such direct 3D scanning of the tools with slim and deep patterns (Figure 9).

Analyzing the results presented in Figure 9, it can be seen that the largest discrepancies/errors are represented by the blue area inside the hole, with the values, even at the bottom, reaching −0.1 mm. In addition, the greater the wear of a given tool, the greater these errors.

In order to better illustrate these differences (in particular in the aspect of forging tool durability), a comparison was made of the volumetric loss for the tool scans and the reversed replication mass scans. The presented results (Figure 9) point to an almost three, and in the case of a new tool, six time difference in the material loss, which all the more confirms the validity of the use of replication masses. In turn, there are no significant relations between the result of the volumetric loss of the dies and their corresponding replication mass imprints. A more detailed analysis of the application of hybrid layers in order to increase the durability of tools confirms that the use of coatings significantly reduces the wear of the dies, of which the BIGAAN coating is more stable, both on the basis of the scanning results (Figure 8) and the comparison of the volumetric loss of the material. In turn, no significant relations were observed of the effect of the material loss size, either on the impression scans or the imprints. Nevertheless, further investigations should be aiming in that direction.

On this basis, we can state that the lack of a full awareness of the effect of the laser beam angle setting in respect of the normal direction to the surface will generate results which strongly differ from the actual state of the matter, and this in turn may result in an improper interpretation of the wear of the tools. What is more, depending on the given tool geometry and the acceptable deviation for the given application, the errors during scanning can be very different and difficult to estimate. This makes it necessary to approach each individual case separately.

## 4. Conclusions

The study discusses the possibilities of applying non-contact 3D scanning techniques for the analysis of the geometrical changes in order to analyze the durability of forging dies used for the forward extrusion of exhaustion valves assigned for motor truck engines. Due to a relatively complicated shape (a slim and deep cavity of the impression) of the analyzed forming tools, the 3D scanning methods may turn out insufficient or burdened with an additional excessive measurement error. That is why some possibilities were proposed for aiding the measurement of this type of inner tool surface with the use of 3D scanning of replication mass imprints of the forging dies’ working impressions. The approach presented by the authors, based on the obtained results, is a fully justified and verified measurement method, which makes it possible to evaluate the actual state of the surface with a sufficient, measurement accuracy for this application (precision forging through forward extrusion).

**Figure 10:**
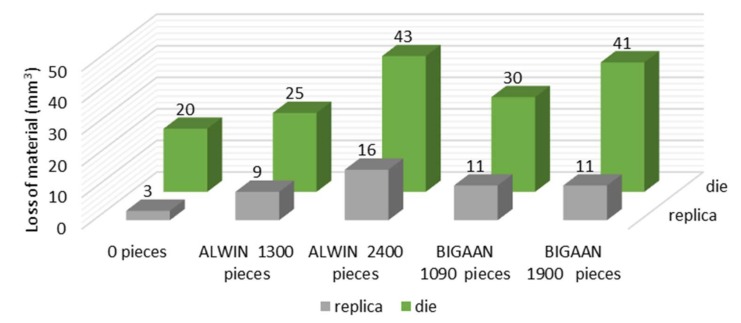
Results of the comparison of the volumetric loss calculated with respect to a new tool for: a new tool scan (illustrating the errors originating from 3D scanning), direct scans of two pairs of tools covered with different coatings and their corresponding reversed 3D replica scans.

The investigation results also show that, during the use of non-contact 3D scanning methods for the measurement of complicated inner cavities of this type, for each particular geometry, the key linear scanning parameters have to be adjusted and verified individually. This is mostly connected with the selection of the optimal angle (between the laser beam and the nominal direction to the scanned surface), in order to possibly cover the whole measurement area, and, at the same time, being aware of the fact that increasing its value drastically increases the measuring error. On this basis, we can draw the following conclusions:

1. For the presented application, the obtained 3D scanning results have shown a satisfactory accuracy for the acceptable angle value of the scanner light reflection equaling 40°, for which the generated errors are acceptable in the case of tool wear analysis. However, in such a case, there is no physical possibility to measure the whole measurement area of interest (deep cavity).
2. For the standard settings of the acceptable angle value (55°) during the process of direct 3D scanning of the dies, the obtained results have confirmed that, based on the colored maps of deviations (scans presented in the first column in Figure 9) as well as the volumetric loss presented in the diagram (Figure 10), the results are burdened with excessive errors, which makes the proper interpretation of tool wear impossible.
3. The analysis of the imprint’s direct 3D scanning results as well as its reversed scan (results presented in the third and fourth column in Figure 9) makes it possible to evaluate the actual degree of wear and thus creates a possibility of a reliable decision about the further operation.
4. The utility of the proposed solution should also be emphasized, as the use of 3D scanning of replication mass imprints for elements of this type will not only enable an evaluation of their actual state (minimizing the errors generated during direct scanning of die impressions), but it will also make it possible to perform non-destructive tests on tools (from the end of the production series) which have produced a relatively small number of forgings. Also, 3D scanning is predisposed to the measurements of impressions from flexible replication masses, due to the lack of contact during the measurement.
